# Perioperative probiotics attenuates postoperative cognitive dysfunction in elderly patients undergoing hip or knee arthroplasty: A randomized, double-blind, and placebo-controlled trial

**DOI:** 10.3389/fnagi.2022.1037904

**Published:** 2023-01-05

**Authors:** Lin Hu, Manli Luo, Huifan Huang, Lanping Wu, Wen Ouyang, Jianbin Tong, Yuan Le

**Affiliations:** ^1^Department of Anesthesiology, The Third Xiangya Hospital, Central South University, Changsha, China; ^2^Hunan Province Key Laboratory of Brain Homeostasis, The Third Xiangya Hospital, Central South University, Changsha, China

**Keywords:** probiotics, postoperative cognitive dysfunction (POCD), elderly patients, cognitive function, hip or knee arthroplasty

## Abstract

**Background:**

Postoperative cognitive dysfunction (POCD) is a common complication in elderly patients following surgery. The preventive and/or treatment strategies for the incidence remain limited.

**Objective:**

This study aimed to investigate the preventive effect of perioperative probiotic treatment on POCD in elderly patients undergoing hip or knee arthroplasty.

**Methods:**

After obtaining ethical approval and written informed consent, 106 patients (age ≥60 years) were recruited, who scheduled elective hip or knee arthroplasty, from 16 March 2021 to 25 February 2022 for this randomized, double-blind, and placebo-controlled trial. They were randomly assigned with a 1:1 ratio to receive either probiotics or placebo treatment (four capsules, twice/day) from hospital admission until discharge. Cognitive function was assessed with a battery of 11 neuropsychological tests on the admission day and the seventh day after surgery, respectively.

**Results:**

A total of 96 of 106 patients completed the study, and their data were finally analyzed. POCD occurred in 12 (26.7%) of 45 patients in the probiotic group and 29 (56.9%) of 51 patients in the placebo group (relative risk [RR], 0.47 [95% confidence interval [CI], 0.27 to 0.81]; *P* = 0.003). Among them, mild POCD occurred in 11 (24.4%) in the probiotic group and 24 (47.1%) in the placebo group (RR, 0.52 [95% CI, 0.29 to 0.94]; *P* = 0.022). No significant difference in severe POCD incidence was found between the two groups (*P* = 0.209). Compared with the placebo group, the verbal memory domain cognitive function was mainly improved in the probiotic group.

**Conclusion:**

Probiotics may be used perioperatively to prevent POCD development and improve verbal memory performance in elderly patients receiving hip or knee arthroplasty.

**Clinical trial registration:**

www.chictr.org.cn, identifier: ChiCTR2100045620.

## 1. Introduction

Postoperative cognitive dysfunction (POCD), characterized by memory, attention, and executive ability impairment (Hood et al., 2018), is highly prevalent in the elderly following orthopedic surgery and is associated with poor clinical outcomes and worst quality of life (Moller et al., 1998; Needham et al., 2017; Deiner et al., 2021). Preventive and/or treatment strategies for POCD development included cognitive and physical exercise (O'Gara et al., 2020; Duan et al., 2022), appropriate depth of anesthesia (Chan et al., 2013), goal-directed fluid therapy (Zhang et al., 2018), effective postoperative analgesia (Kristek et al., 2019), and pharmacologic interventions [e.g., edaravone (Zhang et al., 2020), methylene blue (Deng et al., 2021), dexmedetomidine (Su et al., 2016), and stains (Alam et al., 2018)]. The incidence of POCD in orthopedic patients remains as high as 24.6–75% (Rodriguez et al., 2005; Koch et al., 2007; Ji et al., 2013; Li et al., 2019); thus studying new preventive strategies is urgently needed.

Probiotics are widely used in public and clinically for general health supplements and disease conditions to improve immune function and brain function (Mohajeri et al., 2018; Suez et al., 2019). Recent studies have performed a new insight into the effect of probiotics on postoperative brain function because probiotics have the potential anti-inflammatory capabilities (Zhan et al., 2018; Jiang et al., 2019) and reduce levels of systemic pro-inflammatory cytokines (such as IL-1β, TNF-α, IL-6, IL-10, and IFN-γ) (Schachter et al., 2018; Choi et al., 2020). Our previous study showed that perioperative probiotic treatment has an anti-inflammatory effect and could prevent postoperative cognitive impairment development assessed with Mini-Mental State Examination (MMSE) in the elderly following non-cardiac surgery (Wang et al., 2021). However, MMSE is a broad screening tool and is commonly criticized for its low sensitivity in the diagnosis of POCD. The score of MMSE can be influenced by the education level, leading to false positive indications when patients with a low level of education, or false negative indications when patients with a high level of education (Newman et al., 2007; Malek-Ahmadi et al., 2012). A neuropsychological test battery is wildly recommended to improve study quality (Evered et al., 2018; Borchers et al., 2021). In this study, we further carried out this randomized, double-blind, placebo-controlled trial to investigate the preventive effect of perioperative administration of probiotics on POCD incidence in the elderly undergoing hip or knee arthroplasty using comprehensive neuropsychological battery tests as a cognitive evaluation tool.

## 2. Methods

### 2.1. Study design and participants

This prospective trial protocol was established with the compliance of the CONSORT Statement, approved (R21010) by the Ethics Committee, Third Xiangya Hospital, Central South University, Changsha, China, and registered in the Chinese Clinical Trial Registry (ChiCTR2100045620). After the written informed consent was obtained, elderly patients (age ≥60 years) admitted to the Department of Orthopedics, Third Xiangya Hospital, from 16 March 2021 to 25 February 2022, who met the inclusion criteria, were enrolled and randomly assigned to receive either probiotics or placebo (four capsules, twice/day) during the whole hospitalization period. The incidence of POCD was evaluated by a battery of 11 neuropsychological tests on the admission day and the seventh day after surgery.

**Figure 1:**
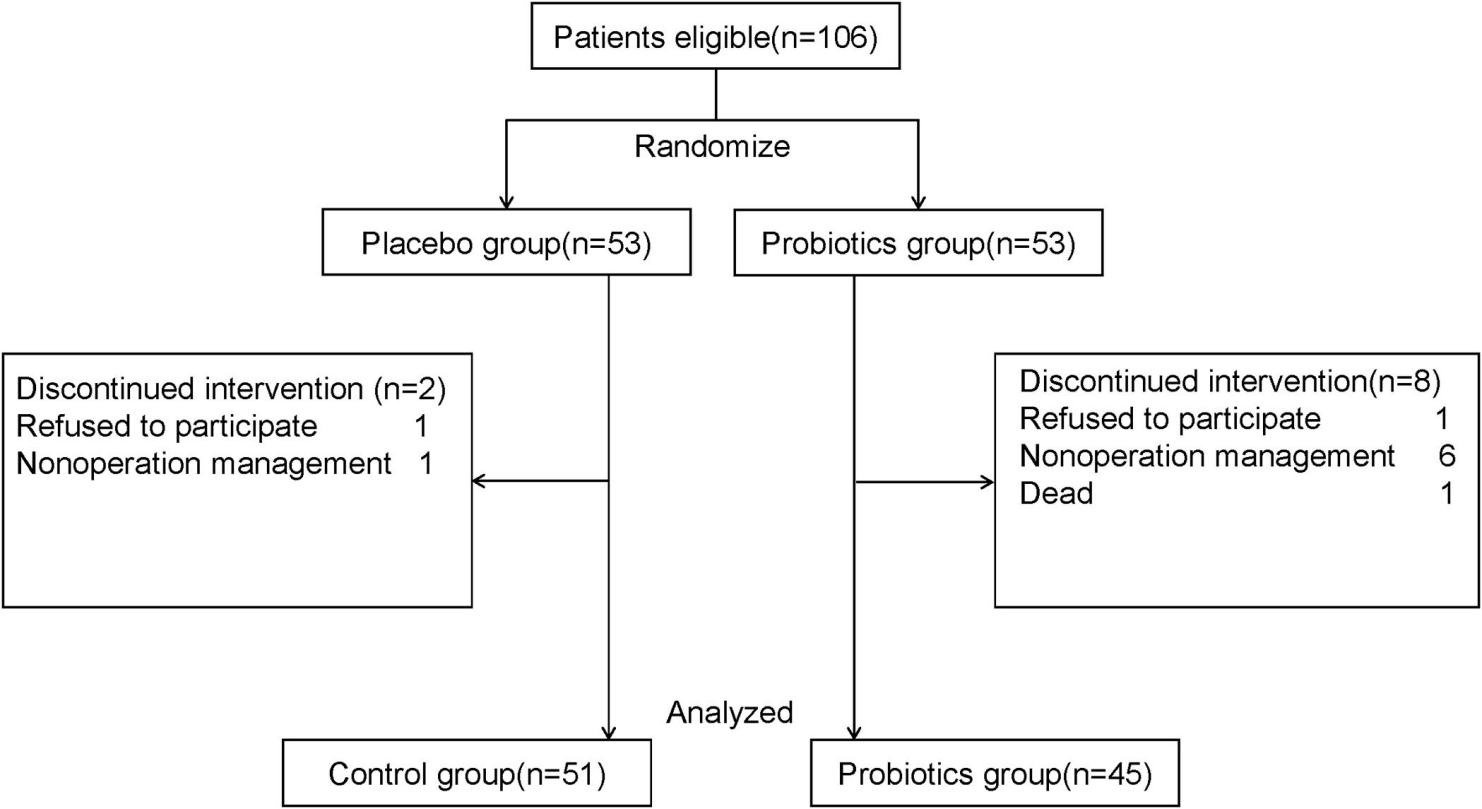
Flow chart of study: Participant eligibility and enrollment are performed.

Patients, who had no history of immune system diseases, psychiatric diseases, and neurodegenerative diseases and were scheduled for elective hip or knee arthroplasty, were eligible to be enrolled in this trial (Figure 1). Patients were excluded when they met any of the following criteria: (1) history of communication disorders (such as severe impairment in speaking, hearing, and vision); myocardial infarctions or poor cardiac function; cerebral hemorrhage, cerebral infarction, brain tumor, and stroke; (2) received more than one surgery during hospital stay; (3) used antibiotics, probiotics, or gastro dynamic drug within 10 days before admission; (4) used complete postoperative parenteral nutrition; (5) mental illness or family history of mental illness; alcoholic or drug addicts; (6) postoperative hospital stay duration was < 7 days; (7) participating in other clinical trials; refuse to join the study; not cooperative with the treatment; and (8) for any other reason that is not suitable for this study.

### 2.2. Blinding and treatment allocation

Patients were randomly divided into the probiotic or placebo groups with concealed allocation by generating random numbers in a 1:1 ratio with SPSS 25.0. Patients and clinicians including surgeons and anesthesiologists and all researchers for pre- and postoperative assessments and data collection were blinded with the trial protocol. However, doctors, who closely looked after patients, can request the unmasking of the treatment assignment or terminate patients' participation if the condition of patients was needed.

### 2.3. Interventions

Patients were randomly assigned to receive either probiotic or placebo treatment (four capsules, twice a day) during the whole hospitalization period and underwent a battery of 11 neuropsychological tests on the admission day and the seventh day after surgery. The probiotic group received four probiotic capsules (0.84 g) twice a day, from hospital admission until discharge. Each probiotic capsule (BIFICO, Sine Pharmaceuticals, Shanghai, China) contained *Bifidobacterium longum* (>10^7^ colony-forming unit [CFU]/210mg)*, Lactobacillus acidophilus* (>10^7^CFU/210 mg), and *Enterococcus faecalis* (>10^7^CFU/210 mg). The placebo capsule (also provided by Sine Pharmaceuticals) contained all ingredients except probiotics with an identical size, shape, and smell as probiotic capsules were given to patients in the placebo group in the same way during hospitalization. To fully guarantee the medical treatment and safety of the patients, we did not limit the other clinical treatments of patients.

### 2.4. Cognitive function assessment

All patients were subjected to cognitive assessments with a battery of 11 neuropsychological tests on the hospital administration (baseline) and the seventh day after surgery by the same assessor who was specifically trained by psychiatrists. The battery tests included the following: Hopkins verbal learning test-revised, delayed recall test, and discrimination index for verbal memory (Lacritz and Cullum, 1998); brief visuospatial memory test-revised (BVMT-R), BVMT-R delayed recall test, and BVMT-R discrimination index for visuospatial memory (Tam and Schmitter-Edgecombe, 2013); number connection test and Benton judgment of line orientation for visuospatial abilities and spatial orientation (Amodio et al., 1999; Boeve et al., 2012); digit span test for attention (Leung et al., 2011); digit symbol substitution test and verbal fluency test for executive function (Jaeger, 2018; Sutin et al., 2019; Juan et al., 2022).

Mild POCD and severe POCD were defined as a decrease of 1–2 standard deviation (SD) or more than 2 SD of two or more neuropsychological tests from the admission baseline to the seventh day after surgery (Sachdev et al., 2014; Borchers et al., 2021). The specific value of the standard deviation is shown in Supplementary Table S1. The change scores of each neuropsychological test were calculated by subtracting the postoperative score from the preoperative score, except for the number connection test in which the change scores were defined as a postoperative score minus the baseline score.

### 2.5. Outcomes and data collection

The primary outcome was the incidence of POCD on the seventh day after surgery. The secondary outcomes included the length of hospital stay, the incidence of hospital death, and 30-day post-hospital death.

The patients' clinical characteristics including laboratory measurements and parameters during anesthesia and surgery including demographics, such as age, sex, height, weight, education, body mass index (BMI), type of operation, American Society of Anesthesiologists (ASA) classification, intraoperative blood loss, type of anesthesia, length of operation, total intraoperative infusion, and type of antibiotics, were collected.

### 2.6. Sample size

The sample size was determined assuming a POCD rate of 40% in the placebo group and 20% in the probiotic group. The POCD rate of 40% in the control group was based on previous studies (Rodriguez et al., 2005; Wu et al., 2018). Given a significance set at the level of 0.5, power at 70%, and a loss to follow-up rate of 10%, a total of 106 patients (*n*_1_= *n*_2_ = 53) are required to detect a difference, according to the formula as follows:


$$n_{1}=\;n_{2}=\;\frac{{\left[Z_{\alpha }\sqrt{2\bar{p}\left(1-\bar{p}\right)}+Z_{\beta }\sqrt{p_{1}\left(1-p_{1}\right)+p_{2}\left(1-p_{2}\right)}\right]}^{2}}{{\left(p_{1}-p_{2}\right)}^{2}}$$

*n*_1_ and *n*_2_ represent the sample size of two groups, *Z*_α_ and *Z*_β_ represent the standard normal deviate values of α and β, and *P*_1_ and *P*_2_ represent the incidence of two groups, $\bar{P}$ = (*P*_1+_ *P*_2_)/2.

### 2.7. Statistical analyses

Normality was tested with the Shapiro–Wilk test. Patients' general characteristics were presented as mean ± standard deviation (SD), or number and percentage, or the median and interquartile range wherever appropriate. Quantitative data with a normal distribution were presented as mean ± standard deviation (SD), or otherwise as the median and interquartile range. Qualitative variables were analyzed with Pearson's chi-square test or Fisher's exact test, and quantitative data were analyzed with a *t*-test or Mann–Whitney U-test where appropriate. The per-protocol (PP) population consisted of all patients who completed the study according to the protocol (Seino et al., 2014). The primary analysis was based on the PP population. The intention-to-treat (ITT) analyses included all randomized patients (Supplementary Tables S2–S6) (Sun et al., 2018). The missing data were calculated using the last observation carried forward imputation method (Lv et al., 2015). All statistical analyses were performed with SPSS software (version 25.0, SPSS, Chicago, United States).

## 3. Results

### 3.1. General characteristics of patients studied

A total of 106 patients were enrolled and randomly assigned to receive either probiotic (*n* = 53) or placebo (*n* = 53) treatment; of those, two patients (2 of 53[3.7%] in the placebo group) and eight patients (8 of 53[15.1%] in the probiotic group) were excluded for various reasons, including refusal to continue participating or cancelation of operations, or one death in the probiotic group; the data from 96 patients were included in the final data analysis (*n* = 51 in the placebo group; *n* = 45 in the probiotic group) (Figure 1). There were no statistical differences in the demographics and clinical characteristics of patients (Table 1) and their baseline scores of neuropsychological tests (Table 2) between the two groups.

**Table 1:** Basic characteristics.

	**Placebo (*n =* 51)**	**Probiotics (*n =* 45)**	***P*-value**
Age (yr)	70 (64,75)	68 (65,73.5)	0.64
Sex			0.55
Male	20 (39.2)	15 (33.3)	
Female	31 (60.8)	30 (66.7)	
Height (cm)	159.76 ± 8.58	159.69 ± 7.11	0.96
Weight (kg)	61.16 ± 10.30	59.90 ± 9.20	0.53
BMI (kg/m^2^)	23.97 ± 3.69	23.46 ± 3.06	0.47
Statins	1 (1.96)	2 (4.4)	0.91
Type of operation			0.61
Knee arthroplasty	23 (45.1)	18 (40)	
Hip arthroplasty	28 (54.9)	27 (60)	
ASA classification			0.203
II	26 (51.0)	16 (35.6)	
III	25 (49.0)	28 (62.2)	
IV	0	1 (2.2)	
Education			0.132
Illiteracy	4 (7.8)	7 (15.6)	
Elementary school	21 (41.2)	15 (33.3)	
Middle school	13 (25.5)	5 (11.1)	
High school	11 (21.6)	12 (26.7)	
University	2 (3.9)	6 (13.3)	

BMI, body mass index; ASA, American Society of Anesthesiologists. All data are presented as number (%), median (first quartile, third quartile), or mean ± SD.

**Table 2:** Results of neuropsychological assessment at baseline.

	**Placebo (*n =* 51)**	**Probiotics (n =45)**	***P*-value**
HVLT-R	10.02 ± 3.11	9.29 ± 3.79	0.30
HVLT-R delayed recall test	2 (1,3)	2 (0,3.5)	0.78
HVLT-R discrimination index	20 (18,22)	20 (17.5,22)	0.78
BVMT-R	6 (3,10)	5 (3.5,11)	0.99
BVMT-R delayed recall test	2 (1,3)	2 (1,4)	0.79
BVLT-R discrimination index	11 (10,12)	10 (10,12)	0.10
Number connection test	431 (360,502)	427 (322,534)	0.83
Benton judgment of line orientation	14 (12,16)	14 (12,15)	0.57
Digit span test	16.29 ± 3.05	16.31 ± 3.92	0.98
Digit Symbol Substitution Test	16 (11,22)	15 (12,24)	0.87
Verbal fluency test	39.84 ± 10.67	39.33 ± 11.41	0.82

HVLT-R, Hopkins verbal learning test-revised; BVMT-R, brief visuospatial memory test-revised. All data are presented as number (%), median (first quartile, third quartile), or mean ± SD.

### 3.2. Parameters during anesthesia and surgery

Parameters during anesthesia and surgery, including operating time, intraoperative infusion volume, intraoperative blood loss, type of anesthesia, postoperative analgesia regime, perioperative dexmedetomidine use, and intraoperative and postoperative antibiotic treatment, were not statistically different between the placebo and probiotic groups (*P* > 0.05, Table 3).

**Table 3:** Parameters during anesthesia and surgery.

	**Placebo (*n =* 51)**	**Probiotics (*n =* 45)**	***P*-value**
Intraoperative blood loss (ml)	100 (50,200)	100 (50,200)	0.06
Total intra-operative infusion (ml)	1,200 (1,100, 1,700)	1,300 (1,050,1,700)	0.69
Dexmedetomidine	15	12	0.77
Type of anesthesia			0.36
intravertebral	27 (52.9)	28 (62.2)	
General	24 (47.1)	17 (37.8)	
Length of operation (h)	120.0 (110.0,155.0)	125.00 (95.0,145.0)	0.74
Intra-operative antibiotic			1
β-lactam	47 (92.2%)	42 (93.3%)	
Quinolones	1 (1.1%)	1 (0.9%)	
Polypeptide	3 (2.7%)	2 (2.3%)	
Postoperative antibiotic			0.87
β-lactam	47 (92.2%)	40 (88.9%)	
Quinolones	1 (2.0%)	2 (4.4%)	
Polypeptide	3 (5.9%)	3 (6.7%)	
Postoperative analgesia regime			0.21
Sufentanil	30 (58.8%)	32 (71.1%)	
Sufentanil and dezocine	21 (41.2)	13 (28.9%)	

All data are presented as number (%), median (first quartile, third quartile), or mean ± SD.

### 3.3. Probiotics decreased the incidence of POCD following surgery

Postoperative cognitive dysfunction occurred in 12 (26.7%) of 45 patients in the probiotic group and 29 (56.9%) of 51 patients in the placebo group (RR, 0.47 [95% CI, 0.27 to 0.81]; *P* = 0.003, Table 4). Among them, mild POCD occurred in 11 (24.4%) in the probiotic group and 24 (47.1%) in the placebo group (RR, 0.52 [95% CI, 0.29 to 0.94]; *P* = 0.022, Table 4). No significant differences in severe POCD incidence were found between the two groups (*P* = 0.209). The mild or severe decline of score mainly occurred in the Hopkins verbal learning test-revised test, Hopkins verbal learning test-revised delayed recall test, and Hopkins verbal learning test-revised discrimination index test in two groups (Table 5).

**Table 4:** Probiotics decrease the incidence of POCD in elderly patients after joint arthroplasty.

**Incidence of cognitive impairment, No./Total (%)**	**Placebo (*n =* 51)**	**Probiotics (*n =* 45)**	**RR (95% CI)**	***P*-value**
Total	29/51 (56.9)	12/45 (26.7)	0.47 (0.27–0.81)	0.003**
Mild	24/51 (47.1)	11/45 (24.4)	0.52 (0.29–0.94)	0.022*
Severe	5/51 (9.8)	1/45 (2.2)	0.23 (0.03–1.87)	0.209

All data are presented as number (%), median (first quartile, third quartile), or mean ± SD; *p < 0.5, **p < 0.01.

**Table 5:** Incidence of mild and severe decline in each neuropsychological test in different groups.

**Incidence of mild and severe decline, No./Total (%)**	**Mild decline**			**Major decline**		
	**Placebo (*n =* 51)**	**Probiotics (*n =* 45)**	***P*-value**	**Placebo (*n =* 51)**	**Probiotics (*n =* 45)**	***P*-value**
HVLT-R	17/51 (33.3)	5/45 (11.1)	0.01*	2/51 (3.9)	1/45 (2.2)	1.000
HVLT-R delayed recall test	20/51 (39.2)	6/45 (13.3)	0.004**	0	0	
HVLT-R discrimination index	8/51 (15.7)	3/45 (6.7)	0.166	9/51 (17.6)	1/45 (2.2)	0.018*
BVMT-R	6/51 (11.8)	5/45 (11.1)	0.920	0	0	
BVMT-R delayed recall test	9/51 (17.6)	7/45 (15.6)	0.784	1/51 (2.0)	1/45 (2.2)	1.000
BVLT-R discrimination index	6/51 (11.8)	4/45 (8.9)	0.746	5/51 (9.8)	3/45 (6.7)	0.719
Number connection test	0	1/45 (2.2)	0.469	0	0	
Benton judgment of line orientation	9/51 (17.6)	7/45 (15.6)	0.784	1/51 (2.0)	0	1.000
Digit span test total	4/51 (7.8)	3/45 (6.7)	1.000	1/51 (2.0)	1/45 (2.2)	1.000
Digit symbol substitution test	4/51 (7.8)	2/45 (4.4)	0.681	0	0	
Verbal fluency test	6/51 (11.8)	6/45 (13.3)	0.817	5/51 (9.8)	0	0.058

HVLT-R, Hopkins verbal learning test-revised; BVMT-R, brief visuospatial memory test-revised. All data are presented as number (%), median (first quartile, third quartile), or mean ± SD; *p < 0.5, **p < 0.01.

### 3.4. Probiotics improve performance in verbal tests

To determine which domain of brain function was mainly improved by probiotics, we further compared the incidence of mild and severe decline in each neuropsychological test between the probiotic and placebo groups. The result showed that compared to the placebo group, the incidence of the mild decline of the Hopkins verbal learning test-revised test (11.1 vs. 33.3%, *P* = 0.01) and the Hopkins verbal learning test-revised delayed recall test (13.3 vs. 39.2%, *P* = 0.004) in the probiotic group was significantly lower (Table 5). The incidence of severe decline of the Hopkins verbal learning test-revised test discrimination index in the probiotic group was also lower than that in the placebo group (2.2 vs. 17.6%, *P* = 0.018). These results suggested that probiotics may improve performance mainly in verbal memory.

### 3.5. Other clinical outcomes

The length of hospital stays, the incidence of hospital death and 30-day post-hospital death, the level of C reactive protein and leukocytes, and the neutrophil percentage did not differ significantly between the placebo and probiotic groups (*P* > 0.05, Table 6).

**Table 6:** Other clinical outcomes.

	**Placebo (*n =* 51)**	**Probiotics (*n =* 45)**	***P*-value**
Length of hospital stays (d)	13 (9,15)	11 (10,14)	0.46
Incidence of hospital death	0	0	1
Incidence of 30-days post-hospital death	0	0	1
**Leukocyte (x10** ^**9**^ **/L)**
Baseline	6.28 (4.56,7.96)	6.05 (5.28,7.24)	0.88
Postoperative day 1	9.95 (2.90)	9.85 (2.55)	0.86
**C reactive protein (mg/L)**
Baseline	5.00 (5.00,16.8)	6.64 (5.00,41.97)	0.23
Postoperative day 1	52.11 (22.97,64.19)	47.97 (30.97,77.65)	0.89
**Neutrophils (%)**
Baseline	62.61 (11.80)	63.47 (13.20)	0.74
Postoperative day 1	81.50 (78.10,87.40)	81.00 (78.50,87.85)	0.85
**Neutrophil count (x10** ^**9**^ **/L)**
Baseline	3.91 (2.78,5.86)	4.00 (3.02,5.23)	0.83
Postoperative day 1	8.19 (2.76)	8.12 (2.33)	0.89

All data are presented as number (%), median (first quartile, third quartile), or mean ± SD.

## 4. Discussion

In the current randomized, double-blind, and placebo-controlled trial, perioperative probiotic treatment significantly reduced the incidence of POCD in patients who underwent elective hip or knee arthroplasty, which is in line with our previous study of the preventive effect of probiotics on POCD development assessed with MMSE (Wang et al., 2021). The length of hospital stays, the incidence of hospital death and 30-day post-hospital death, the level of C reactive protein, and blood cell counts were not significantly different between the placebo and probiotic groups. Furthermore, ITT analyses yielded the same conclusions (Supplementary Tables S2–S6). Our study suggests that perioperative probiotic supplements may be potential strategies for preventing POCD development in elderly patients.

Previous studies reported that perioperative peripheral inflammatory responses act as a major mechanism in the pathogenesis of POCD *via* inducing neuroinflammation and, as a result, damaging synapses connectivity (Tanabe et al., 2020; Zhu et al., 2021; Chen et al., 2022) and triggering cognitive decline. Limiting perioperative peripheral inflammatory responses may significantly alleviate POCD in the pre-clinical setting (Cibelli et al., 2010; Terrando et al., 2010). Pharmacologic interventions such as anti-inflammatory drugs, dexmedetomidine (Su et al., 2016), and statins (Alam et al., 2018) have also been reported to have certain effects in preventing POCD clinically. However, preventing POCD remains a clinical challenge. Accumulating evidence showed that gut microbial dysbiosis can affect peripheral inflammation (Fung et al., 2017), the pathogenesis of psychological diseases (Cryan et al., 2019), neurodegenerative diseases (Sun and Shen, 2018), and cognitive impairment following surgery (Xu et al., 2020). Probiotic supplements can significantly alleviate gut microbial dysbiosis and its related pathological effects (O'Mahony et al., 2005; Chunchai et al., 2018). Previous studies also demonstrated that gut microbial dysbiosis promoted peripheral inflammatory response *via* damaging the intestinal wall, changing peripheral metabolites' levels, and modulating HPA axis response (Fung et al., 2017), while probiotic supplements negated all these changes effectively (Suez et al., 2019; Juan et al., 2022). It has been reported that antibiotic administration affects the intestinal microbiota (Dethlefsen and Relman, 2011), which may result in antibiotic-related diarrhea and intestinal complications, such as *Clostridium difficile*-related colitis (De La Cochetière et al., 2005). To exclude the effects of antibiotics on the composition of gut microbiota, subjects who reported antibiotics treatments 10 days before admission were excluded (Wang et al., 2021). No differences were found with respect to the type of intraoperative antibiotic and the type of postoperative antibiotic between the two groups. In this study, we found that perioperative probiotic supplements significantly reduced the incidence of POCD in elderly patients, which further supports our previous findings (Wang et al., 2021) and provides a new strategy for preventing POCD in elderly patients. Further mechanistic investigation showed that perioperative probiotic supplements accelerated the postoperative decrease of inflammatory cytokines and glucocorticoids in peripheral blood (Wang et al., 2021). It is likely that probiotic supplements offered multi-benefits to our surgical patients, and the underlying mechanisms need to be studied further.

Our study had several limitations. First, as a single-center study with a small sample size and simple surgical population, the enrolled patients may not fully represent the patient population. Second, long-term follow-up was not done, and hence whether the treatment improves long-term outcomes remain unknown. Third, the underlying mechanism for the prevention of POCD using probiotic supplements remains elusive.

## 5. Conclusion

Our study indicated that a convenient perioperative supplement of probiotics can effectively mitigate postoperative cognitive impairment and improve performance mainly in verbal memory in elderly patients following hip or knee arthroplasty. Furthermore, a large sample-size trial is needed before the strategy can be used clinically to tackle the development of POCD.

## Data availability statement

The raw data supporting the conclusions of this article will be made available by the authors, without undue reservation.

## Ethics statement

The studies involving human participants were reviewed and approved by the Ethical Committee of the Third Xiangya Hospital of Central South University, China. The patients/participants provided their written informed consent to participate in this study.

## Author contributions

YL designed the study. LH, ML, HH, and LW conceived the original data. LH and HH performed the statistical analysis. LH wrote the manuscript. YL, WO, and JT reviewed the manuscript. All authors agree to be accountable for the content of the work.
