# Supplementary material for: Perioperative probiotics attenuates postoperative cognitive dysfunction in elderly patients undergoing hip or knee arthroplasty: A randomized, double-blind, and placebo-controlled trial
Source: Front Aging Neurosci. 2023 Jan 5;14:1037904. doi: 10.3389/fnagi.2022.1037904 (PMC9849892; doi:10.3389/fnagi.2022.1037904)
Supplement: Supplementary file 1 [file Data_Sheet_1.docx]

| Table S1. The specific value of SD in each cognitive test |  |
| --- | --- |
| Cognitive test | SD |
| HVLT-R | 3.45 |
| HVLT-R delayed recall test | 1.60 |
| HVLT-R discrimination index | 2.61 |
| BVMT-R | 4.01 |
| BVMT-R delayed recall test | 1.65 |
| BVLT-R discrimination index | 1.43 |
| Number connection test | 178.77 |
| Benton judgment of line orientation | 2.72 |
| Digit span test | 3.47 |
| Digit Symbol Substitution Test | 9.46 |
| Verbal fluency test | 10.97 |
| SD: Standard deviation. |  |

| Table. S2 ITT analysis of basic characteristics | | |  |  |
| --- | --- | --- | --- | --- |
|  | Placebo (n = 53) | Probiotics (n = 53) | *P* value |  |
| Age(yr) | 70(59,81) | 69(60,78) | 0.98 |  |
| Sex |  |  | 0.84 |  |
| Male | 20(37.7) | 19(35.8) |  |  |
| female | 33(62.3) | 34(64.2) |  |  |
| Height (cm) | 158(145.5,170.5) | 159(149,169) | 0.64 |  |
| Weight (kg) | 61.34±10.14 | 58.67±9.64 | 0.80 |  |
| BMI (kg/m²) | 24.13±3.73 | 23.00±3.26 | 0.4 |  |
| Statins | 1(1.9) | 2(3.8) | 0.91 |  |
| Education |  |  | 0.22 |  |
| illiteracy | 4(7.5%) | 7(13.2%) |  |  |
| elementary school | 21(39.6%) | 16(30.2%) |  |  |
| middle school | 14(26.4%) | 10(18.9%) |  |  |
| high school | 12(22.6%） | 12(22.6%） |  |  |
| university | 2(3.8%) | 8（15.1%) |  |  |
| BMI: body mass index; ASA: American Society of Anesthesiologists. All data are presented as number (%), median (first quartile, third quartile) or mean ± SD. | | | |  |
|  |  |  |  |  |

| Table S3. ITT analysis of neuropsychological assessment at baseline | | | |
| --- | --- | --- | --- |
|  | Placebo (n = 53) | Probiotics (n =53) | *P* value |
| HVLT-R | 9.96±3.08 | 9.36±3.53 | 0.35 |
| HVLT-R delayed recall test | 2(1,3) | 2(0,4) | 0.55 |
| HVLT-R discrimination index | 20(18,22) | 20(18,22) | 0.87 |
| BVMT-R | 6(3,10) | 6(4,10) | 0.85 |
| BVMT-R delayed recall test | 2(1,3) | 2(1,4) | 0.91 |
| BVLT-R discrimination index | 11(10,12) | 11(10,12) | 0.14 |
| Number connection test | 431(362,500) | 432(335,529) | 0.89 |
| Benton judgment of line orientation | 14(12,16) | 14(12,15) | 0.46 |
| Digit span test | 16.25±3.01 | 16.42±3.66 | 0.8 |
| Digit Symbol Substitution Test | 16(12,22) | 15(13,22) | 0.94 |
| Verbal fluency test | 39.87±10.47 | 39.40±10.72 | 0.82 |
| HVLT-R: Hopkins verbal learning test-revised; BVMT-R: Brief visuospatial memory test-revised. All data are presented as number (%), median (first quartile, third quartile) or mean ± SD. | | | |

| Table S4. ITT analysis of incidence of POCD. | | | | |
| --- | --- | --- | --- | --- |
| Incidence of cognitive impairment, No./Total (%) | Placebo (n = 53) | Probiotics (n = 53) | RR (95% CI) | P value |
| Total | 30/53(56.6) | 15/53(28.3) | 0.50(0.31-0.82) | 0.003** |
| Mild | 25/53(47.2) | 14/53(26.4) | 0.56(0.33-0.95) | 0.027* |
| Severe | 5/53(9.4) | 1/53(1.9) | 0.20(0.02-1.66) | 0.207 |
| All data are presented as number (%), median (first quartile, third quartile) or mean ± SD; **p*< 0.5, ***p* < 0.01. | | | |  |

| Table S5. ITT analysis of incidence of mild and severe decline in each neuropsychological tests in different groups. | | | | | | |
| --- | --- | --- | --- | --- | --- | --- |
| Incidence of mild and severe decline, No./Total (%) | Mild decline | | | Major decline | | |
|  | Placebo (n = 53) | Probiotics (n = 53) | *P* value | Placebo (n = 53) | Probiotics (n = 53) | *P* value |
| HVLT-R | 17(32.1) | 5(9.4) | 0.004** | 2(3.8) | 1(1.9) | 1 |
| HVLT-R delayed recall test | 20(37.7) | 6(11.3) | 0.002** | 0 | 0 |  |
| HVLT-R discrimination index | 8(15.1) | 3(5.7) | 0.111 | 9(17.0) | 1(1.9) | 0.008** |
| BVMT-R | 4(7.5) | 6(11.3) | 0.506 | 0 | 0 |  |
| BVMT-R delayed recall test | 9(17.0) | 7(13.2) | 0.587 | 1(1.9) | 1(1.9) | 0.475 |
| BVLT-R discrimination index | 6(11.3) | 4(7.5) | 0.506 | 5(9.4) | 3(5.7) | 0.713 |
| Number connection test | 0 | 2(3.8) | 0.475 | 0 | 0 |  |
| Benton judgment of line orientation | 9(17.0) | 7(13.2) | 0.587 | 1(1.9) | 0 | 1 |
| Digit span test total | 4(7.5) | 3(5.7) | 1 | 1(1.9) | 1(1.9) | 0.475 |
| Digit Symbol Substitution Test | 4(7.5) | 2(3.8) | 0.674 | 0 | 0 |  |
| Verbal fluency test | 6(11.3) | 6(11.3) | 1 | 5(9.4) | 0 | 0.067 |
| HVLT-R: Hopkins verbal learning test-revised; BVMT-R: Brief visuospatial memory test-revised. All data are presented as number (%), median (first quartile, third quartile) or mean ± SD; **p*< 0.5, ***p* < 0.01. | | | | | | |

| Table S6. ITT analysis of other clinical outcomes | | |  |
| --- | --- | --- | --- |
|  | Placebo (n = 53) | Probiotics (n = 53) | *P* value |
| Length of hospital stays(d) | 13(9,15） | 11(9.5,14) | 0.471 |
| Incidence of hospital death | 0 | 1(1.9) | 1 |
| Incidence of 30-days post-hospital death | 0 | 1(1.9) | 1 |
| Leukocyte(x10^9^/L) |  |  |  |
| Baseline | 6.28(4.57,7.90) | 6.05(5.34,7.24) | 0.972 |
| Postoperative day 1 | 9.40(7.64,12.14) | 9.55(7.97,11.53) | 0.808 |
| C reactive protein(mg/L) | |  |  |
| Baseline | 5(5,10.58) | 5(5,34.98) | 0.277 |
| Postoperative day 1 | 35.25(16.87,63.65) | 46.81(27.44,77.65) | 0.193 |
| Neutrophils (%) |  |  |  |
| Baseline | 62.13±11.91 | 63.15±15.30 | 0.703 |
| Postoperative day 1 | 81.60(77.80,87.38) | 80.4(76.5,87.85) | 0.919 |
| Neutrophil count(x10^9^/L) | |  |  |
| Baseline | 3.9(2.5,5.78) | 4(3.02,5.20) | 0.766 |
| Postoperative day 1 | 7.64(5.95,10.33) | 7.82(6.13,9.78) | 0.909 |
| All data are presented as number (%), median (first quartile, third quartile) or mean ± SD. | | | |
